# Beyond the Bloom: Unraveling the Diversity, Overlap, and Stability of Free-Living and Particle-Attached Bacterial Communities in a Cyanobacteria-Dominated Hypereutrophic Lake

**DOI:** 10.1007/s00248-024-02410-2

**Published:** 2024-07-24

**Authors:** Guijuan Xie, Chuanbo Sun, Yi Gong, Wenlei Luo, Xiangming Tang

**Affiliations:** 1 College of Biology and Pharmaceutical Engineering, West Anhui University, Lu’an, 237012 China; 2 State Key Laboratory of Lake Science and Environment, Nanjing Institute of Geography and Limnology, Chinese Academy of Sciences, Nanjing, 210008 China; 3 The Fuxianhu Station of Plateau Deep Lake Field Scientific Observation and Research, Yuxi, 653100 Yunnan China; 4 College of Resources and Environment, University of Chinese Academy of Sciences, Beijing, 100049 China

**Keywords:** Organic aggregates, Cyanobacterial bloom, Bacterial diversity, Community composition, Co-occurrence networks, Lake Xingyun

## Abstract

In aquatic ecosystems with low nutrient levels, organic aggregates (OAs) act as nutrient hotspots, hosting a diverse range of microbial species compared to those in the water column. Lake eutrophication, marked by intensified and prolonged cyanobacterial blooms, significantly impacts material and energy cycling processes, potentially altering the ecological traits of both free-living (FL) and particle-attached (PA) bacteria. However, the extent to which observed patterns of FL and PA bacterial diversity, community assembly, and stability extend to hypereutrophic lakes remains understudied. To address this gap, we investigated bacterial diversity, composition, assembly processes, and stability within hypereutrophic Lake Xingyun. Our results revealed that FL bacterial communities exhibited higher α-diversity than PA counterparts, coupled with discernible taxonomic compositions. Both bacterial communities showed distinct seasonality, influenced by cyanobacterial bloom intensity. Environmental factors accounted for 71.1% and 54.2% of the variation among FL and PA bacteria, respectively. The assembly of the PA bacterial community was predominantly stochastic, while FL assembly was more deterministic. The FL network demonstrated greater stability, complexity, and negative interactions, indicative of competitive relationships, while the PA network showed a prevalence of positive correlations, suggesting mutualistic interactions. Importantly, these findings differ from observations in oligotrophic, mesotrophic, and eutrophic lakes. Overall, this research provides valuable insights into the interplay among bacterial fractions, enhancing our understanding of nutrient status and cyanobacterial blooms in shaping bacterial communities.

**Supplementary Information:**

The online version contains supplementary material available at 10.1007/s00248-024-02410-2.

## Introduction

Microorganisms, particularly bacteria, play pivotal ecological roles within aquatic ecosystems [1, 2]. They contribute significantly to nutrient cycling by decomposing organic matter, participate in water purification processes such as bioremediation, and form the foundation of aquatic food webs by serving as primary producers or decomposers [3, 4]. Within aquatic ecosystems, the water column typically harbors a substantial quantity of organic aggregates (OAs) [5]. Enriched with particulate organic matter (POM) and abundant microorganisms, it becomes a hotspot for the mineralization of organic substances and the recycling of nutrients in the water [6–8]. Traditionally, bacteria associated with these OAs are designated as particle-attached (PA) bacteria, while those existing freely in the water column are termed free-living (FL) bacteria [9, 10]. Furthermore, it is hypothesized that numerous aquatic bacteria might undergo an alternating lifestyle, transitioning between FL and PA stages [10]. Many studies indicate that despite distinct differences in composition between PA and FL communities [11–13], there is a clear ecological interconnection between them [14–16]. Both communities are highly responsive to environmental changes, such as lake eutrophication [17, 18]. Therefore, understanding the diversity of these bacteria, the mechanisms governing community assembly, and their responses to environmental changes are fundamental aspects of aquatic microbial ecology.

Due to continuous pollutant discharge and the impact of climate change, eutrophication has become a significant global challenge within aquatic ecosystems, particularly in lakes [19, 20]. Eutrophication can lead to heightened algal density in water, potentially forming harmful algal blooms (e.g., cyanobacterial blooms), and deteriorating water quality. The growth of algae creates a more advantageous setting for the proliferation of attached bacteria, altering the dispersal characteristics of both FL and PA bacteria in the water, and profoundly affecting material and energy cycling processes in lakes [13]. Previous research indicates that in oligotrophic, mesotrophic, and some eutrophic lakes, PA bacteria exhibited a more diverse community than that of FL fraction, with more pronounced differences observed within lakes with lower nutrient levels [21]. Occasional reports suggesting a lower diversity of PA bacteria compared to the FL counterpart may be related to variations in filter membrane pore size, seasonal variations in sampling, or differences in research methodologies [14, 21, 22]. Research has demonstrated that deterministic processes exert a more significant influence than stochastic processes in shaping the assembly of bacterial communities, with deterministic processes influencing PA bacteria more than their FL counterpart [18, 23, 24]. Furthermore, the network analysis showed that the PA bacterial community is more stable compared to the FL fraction [18, 23]. Eutrophication has the potential to decrease FL network complexity while enhancing its stability [25].

These findings underscore the substantial impact of water nutritional status on the diversity, community stability, and community assembly processes of both FL and PA bacteria. With further increases in nutrient levels, particularly in hypereutrophic lakes, the concentration and duration of algal-derived OAs, primarily from cyanobacteria, are elevated [26]. The size, source, and composition of particles are considered crucial factors influencing bacterial communities [14, 27]. This is likely to affect the structure and function of both FL and PA bacterial communities. In this context, whether the observed patterns of FL and PA bacterial diversity, community stability, and assembly processes still hold true in hypereutrophic lakes remains an important and unexplored area of research. Understanding the variations of these two bacterial communities in hypereutrophic lakes is vital to comprehending the overall microbial ecology of aquatic habitats and their roles in ecosystem processes.

To bridge the aforementioned knowledge gap, we carried out the research to explore the spatiotemporal succession patterns exhibited by both FL and PA bacteria in a hypereutrophic lake (Lake Xingyun), aiming to address the following pivotal questions: (1) Which one exhibits higher diversity in PA and FL bacterial assemblages in the hypereutrophic lake? (2) What are the commonalities and variations in the structure, community composition, and driving factors of these two bacterial assemblages? (3) How do the underlying community assembly mechanisms, network characteristics, and community stability differ between the two bacterial assemblages?

## Materials and Methods

### Study Area, Sample Collection, and Environmental Parameter Measurement

**Fig. 1:**
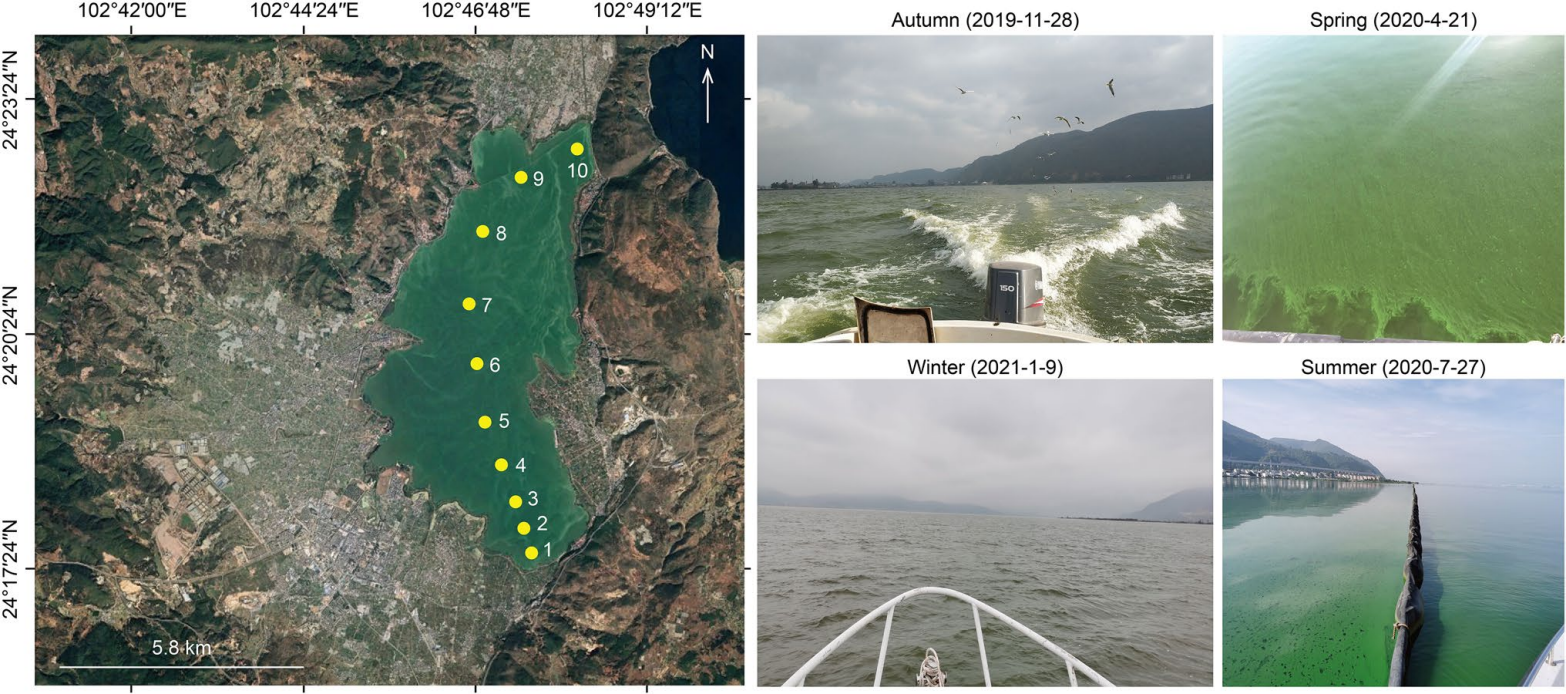
Map depicting the spatial distribution of the 10 sampling sites in Lake Xingyun with photographs of the scene

As one of the nine plateau lakes in Yunnan Province, Lake Xingyun, 24°17′–24°23′ N, 102°45′–102°48′ E, constitutes an integral section of the Nanpan River system within the broader Pearl River basin. The lake area is situated within a subtropical southwestern monsoon climate regime, featuring a mean annual temperature of 15 °C [28]. Annually, the region receives an average precipitation of 947 mm, with a remarkable 80% concentrated during the months spanning May to October. The annual evaporation rate is measured at 1192.3 mm. Covering a catchment area of 378 km^2^ and nourished by 12 inflow rivers, Lake Xingyun rests at an elevation of 1722 m. The water surface extends across an approximate area of 34.7 km^2^, boasting a volumetric capacity of approximately 184 million m^3^. With a mean depth of 5.3 m (maximum depth = 11.0 m), the lake confronts severe water pollution, classified as Class V (worst category) in terms of water quality. Evidently, it is a heavily eutrophic lake, with recurrent cyanobacterial blooms plaguing its water throughout the year. In our investigation, we strategically identified and sampled from 10 sampling sites positioned along an approximately 11-km transect extending from the south to the northeast (Fig. 1).

At each sampling site, approximately 10 l of water was collected from the lake surface (top 50 cm) in triplicate, using a 5-l sampler pre-rinsed with lake water in situ. The sampling period spans four seasons: 28 November 2019 (Autumn), 21 April 2020 (Spring), 27 July (Summer), and 9 January 2021 (Winter). With dark cooling boxes, water samples were carried to the laboratory for immediate chemical analysis. For DNA extraction, subsamples of 300 ml water underwent sequential filtration through 5.0 µm and 0.2 µm polycarbonate filter (Millipore), respectively, enabling the separation of FL bacteria (0.2–5 µm) and PA (≥ 5.0 µm) [21]. The resultant filters were stored at − 80 °C.

At each sampling site’s approximately 50 cm depth, in situ measurements were conducted for pH, water temperature (WT), and electrical conductivity (EC) using a YSI EXO2 multiparameter water quality sonde (USA). Transparency (SD) was assessed with a Secchi disk. Total nitrogen (TN), total phosphorus (TP), total dissolved nitrogen (TDN), total dissolved phosphorus (TDP), dissolved organic carbon (DOC), chlorophyll*-a* (Chl-*a*), chemical oxygen demand (COD), loss of ignition (LOI), and total suspended solids (TSS) were measured using standard methods [29]. Proportion of organic matter (OM), particulate phosphorus (PP), and particulate nitrogen (PN) were calculated using the following formula: OM = (TSS – LOI)/TSS × 100%; PP = TP − TDP; PN = TN − TDN. To evaluate lake trophic states, the comprehensive trophic level index (TLI) was computed following the methodology by Wang et al. [30].

### Characteristics of OA and Bacterial Abundances

To assess the abundances of OA (*A*_OA_), total bacteria (TB), particle-attached bacteria, and free-living bacteria, literature guidance was followed [21, 31]. Moreover, the surface area of each OA was calculated using ImageJ 1.52 software, with particles exhibiting a surface area greater than 5 µm^2^ and clear bacterial attachment being classified as OAs in this study.

### Molecular Analyses

FastDNA® Spin Kit for Soil (MP Biomedicals) was used to extract the genomic DNA of the samples. Bacterial 16S rRNA V3-V4 genes were amplified using the primers 338F and 806R [32]. PCR was conducted as outlined by Xie et al. [21]. Pair-end sequencing (2 × 300 bp) was performed using the platform of Illumina MiSeq PE300 at the Beijing Genomics Institute (BGI), China.

Bioinformatic analysis was conducted using the QIIME2 (Core 2023.2) [33]. A standard analysis protocol, including filtering, denoising, and quality control through the DADA2 plugin was executed [34]. Amplicon sequence variants (ASVs) were generated, and taxonomy classification was achieved using the SILVA v138 database [35]. Sequences belonging to archaea, chloroplast, mitochondria, and unassigned (less than 0.01% of the total sequences) were omitted from further analysis. To mitigate random sequencing errors, ASVs with < 10 reads were excluded. Bacterial *α*-diversity metrics, such as observed ASVs, Shannon index, Faith’s phylogenetic diversity (PD), and Pielou evenness, were calculated using a consistent sequencing depth of 33,669 reads. To evaluate bacterial β-diversity, particularly variations in bacterial community compositions (BCCs) among different fractions (FL vs. PA) and seasons, nonmetric multidimensional scaling (nMDS) analysis, and cluster analysis were conducted, employing Bray–Curtis distance. These analyses were conducted using the PRIMER-E v7 [36].

For the detection of bacterial taxa showing differential abundance between fractions (FL vs. PA), we utilized LEfSe (linear discriminant analysis (LDA) effect size) on ASVs with relative abundance > 0.1%. This analysis was performed using the online Galaxy interface (http://huttenhower.sph.harvard.edu/galaxy) with an LDA threshold score of 4.0 and Kruskal–Wallis *P*-value of 0.01.

### Ecological Processes Analysis of Bacterial Community Assembly

The relative significance of stochastic processes in shaping the assembly of FL and PA bacterial communities was evaluated using the modified stochasticity ratio (MST). MST measures the difference between observed similarity matrices and the expectation from a null model, offering a quantitative measure to evaluate the influence of stochastic factors on variations in BCCs. This analysis was conducted using the R code provided by Ning et al. [37], with the statistical significance of MST determined by bootstrapping using “nst.boot” function in “NST” package.

### Co-occurrence Network Construction and Analysis

To better understand species interactions in community assembly, we utilized network theory [38] to analyze co-occurrence patterns in FL and PA bacterial fractions. ASVs with > 0.05% relative abundance and presence in > 50% samples were selected for network construction. We employed the molecular ecological network analysis (MENA) pipeline (http://ieg4.rccc.ou.edu/mena/), using Pearson correlation coefficient and random matrix theory (RMT) modeling for threshold identification (cutoff = 0.83) [39].

Modules in the networks were identified through fast greedy modularity optimization [40], categorizing nodes (ASVs) into peripheral nodes, connectors, module hubs, and network hubs based on within-module connectivity (*Z*_i_) and among-module connectivity (*P*_i_) [41]. We established a baseline by calculating the topological properties of 100 random networks with equal nodes and edges. Visualizations were created using Gephi (v0.10.1) (http://gephi.org).

Furthermore, bacterial community stability was assessed using the average variation degree (AVD) from the rarefied ASV table, following Xun et al. [42]. Lower AVD values indicate higher microbiome stability. This comprehensive approach enabled us to decipher complex species dynamics and evaluate overall bacterial community stability in the studied environment.

### Statistical Analyses

Statistical analyses were conducted using R 4.2.1 with the RStudio 2022.12.0 interface. Data visualization was performed using the “*ggplot2*” package. Non-parametric Kruskal–Wallis tests were used to examine differences in physicochemical parameters among seasons and bacterial α-diversity between fractions and among seasons, with Holm correction for multiple comparisons. The difference in BCCs among treatments was statistically assessed using analysis of similarity (ANOSIM) based on the bacterial Bray–Curtis similarity distance matrix, employing 999 permutations [43]. Levins niche width [44] between FL and PA bacterial communities was computed using the “*spaa*” (SPecies Association Analysis) package (v0.2.2).

For exploring relationships between environmental factors and BCCs, redundancy analysis (RDA) was conducted with forward selection [45]. ASV data were Hellinger transformed as the independent variable, while environmental parameters served as explanatory variables. Monte Carlo replacement tests, implemented with the “*permutest()*” function, were utilized to assess the significance of environmental factors. Collinear environmental factors with a variance inflation factor (VIF) exceeding 10 were identified and removed using the “*vif.cca()*” function. Lastly, the “rdacca.hp” package evaluated the independent effect of each significant variable on BCC variation [46].

## Results

### Characterizes of Environmental Parameters, OA, and Bacterial Abundances

Substantial seasonal fluctuations in environmental parameters (Supplementary Fig. S1) and those related to OA (Fig. S2) were observed in Lake Xingyun. Interestingly, no significant variations were noted among different sampling sites (*P* > 0.05). WT was highest in summer (average 23.4 °C) and lowest in winter (13.0 °C), while pH reached its highest value in autumn (9.27) and its lowest in spring (8.70) (Fig. S1). SD attained its maximum in autumn (76 cm) and its minimum in summer (42 cm). Notably, the concentrations of TN, TP, COD, Chl-*a*, and TSS were highest during the summer season and considerably lower in autumn and winter. In situ observations indicated that cyanobacterial blooms in Lake Xingyun were dominated by *Microcystis* spp. throughout all four seasons.TLI consistently exceeded 66 in all seasons, peaking at 84 in summer, indicative of the hypereutrophic status of this freshwater lake.

Abundances of OA (A_OA_) ranged from 6 × 10^4^ ind./ml in autumn to 27 × 10^4^ ind./ml in summer, with the average surface area per OA reaching its zenith in autumn (288 µm^2^) and its nadir in spring (95 µm^2^) (Fig. S2). Meanwhile, PAB abundances varied from 0.8 × 10^6^ cells/ml in winter to 13.3 × 10^6^ cells/ml in spring, with the relative abundance of PAB reaching 67% in autumn and hitting its lowest point in summer (23%). From spring to winter, abundances of FL bacteria exhibited a decreasing trend, ranging from 24.4 × 10^6^ to 2.0 × 10^6^ cells/ml. The proportion of OM and PN in TSS peaked in summer at 87% and 81%, respectively, while PP exceeded 56% in all seasons, reaching its highest value of 84% in spring. This suggests a consistently high nutrient ratio (carbon, nitrogen, and phosphorus) within OA in Lake Xingyun, as OA constituted the primary components of TSS.

### Bacterial α- and β-Diversity Across FL and PA Fractions and Seasons

After stringent quality control procedures, a dataset comprising 3,239,107 high-quality reads was obtained (Table S1), with an average length of 419 bp. The average number of reads per sample was 40,489 (ranging from 33,669 to 53,325). These reads were assigned to 1747 ASVs across the 80 samples, taxonomically distributed among 31 phyla, 77 classes, 181 orders, 283 families, and 455 genera.

**Fig. 2:**
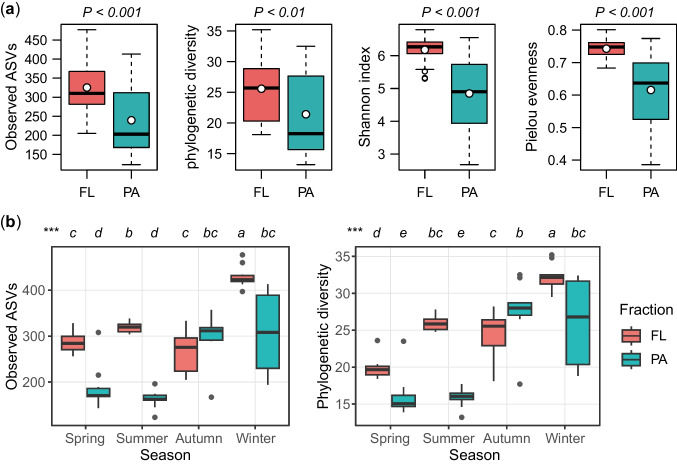
Bacterial α-diversity indices illustrating differences between fractions (**a**) and across seasons (**b**) in Lake Xingyun. Bacterial *α*-diversity indices were calculated using a subset of 33,669 reads per sample. The horizontal black wide lines and hollow circles represent median and mean values, respectively. The upper and lower edges of the boxes indicate the first and third quartiles, with whiskers representing the lowest and highest values within 1.5 times the interquartile range of the lower and quartiles. Kruskal–Wallis test with Holm correction of *P*-values was performed for multiple comparisons to assess differences between fractions and across seasons. Different lower-case letters above each boxplot signify significant differences (*P* < 0.05) among seasons. ***P < 0.001, denoting overall significance. FL, free-living; PA, particle-attached

The rarefaction curves approach an asymptotic maximum once the sequencing depth exceeds 10,000, which suggests that the sequencing depth is adequate to provide a stable and unbiased estimation of α-diversity (Fig. S3). The FL fraction displayed noticeably higher α-diversity indices, such as observed ASVs, PD, Shannon index, and Pielou evenness, compared to the PA fraction (Fig. 2a). These indices showed significant seasonal variations for both FL and PA bacterial communities, with the highest values observed in winter (Fig. 2b, Fig. S4). However, there were no significant differences in α-diversity indices among sampling sites for either FL or PA bacterial assemblages (Fig. S5).

**Fig. 3:**
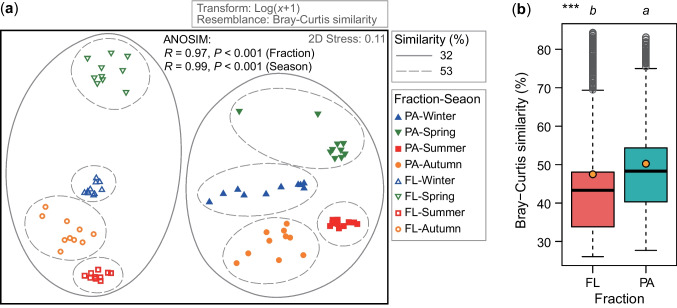
**a** Non-metric multidimensional scaling (NMDS) showing the *β*-diversity of bacterial communities between fractions and across seasons in Lake Xingyun. **b** Comparison of Bray–Curtis similarity between free-living (FL) and particle-attached (PA) bacterial fractions. ***P < 0.001

BCCs displayed notable variations based on fraction (PA vs. FL) and seasons (Fig. 3a). ANOSIM revealed statistically significant differences in BCCs between FL and PA fractions (*P* < 0.001) and among seasons (*P* < 0.001). Cluster analysis demonstrated a distinct separation between FL and PA samples at a Bray–Curtis similarity of 30%, with further separation among samples from different seasons occurring at an increased similarity of 52% for both FL and PA fractions. Seasonal dynamics were more distinct for FL bacteria, as indicated by significantly lower Bray–Curtis similarity in FL samples compared to PA samples (Fig. 3b).

**Fig. 4:**
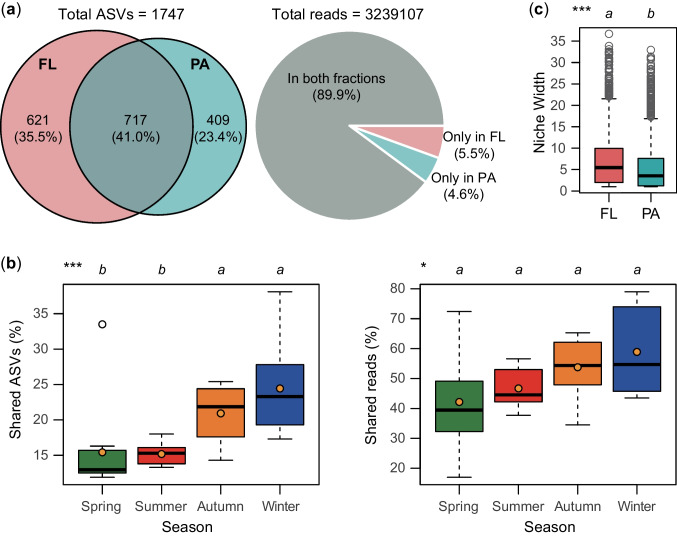
**a** Venn diagram and pie plot presenting the number of amplicon sequence variants (ASVs) and the related reads shared between free-living (FL) and particle-attached (PA) bacterial fractions, respectively. **b** Boxplot showing the percentage of shared ASVs and reads across seasons. Kruskal–Wallis test with Holm correction of *P*-values was performed to evaluate differences among seasons. **c** Comparison of niche width between FL and PA bacterial fractions. *P < 0.05; ***P < 0.001

Among the 1747 ASVs identified in this study, 41.0% were shared between both fractions, while 35.5% and 23.4% were exclusive to FL and PA samples, respectively (Fig. 4a). Accounting for the abundance of each ASV, 89.9% of the reads were shared between both fractions, with 5.5% and 4.6% occurring exclusively in FL and PA fractions, respectively (Fig. 4a). Seasonally, the shared ASV percentages in autumn (21%) and winter (24%) were significantly higher compared to spring (15%) and summer (15%) (Fig. 4b). Additionally, the proportion of shared reads increased from spring to winter, ranging from 42 to 59% (Fig. 4b). Niche width was significantly lower in the PA fraction than in the FL counterpart (*P* < 0.001; Fig. 4c).

### Bacterial Taxonomy Variations Within Fractions Across Different Seasons

**Fig. 5:**
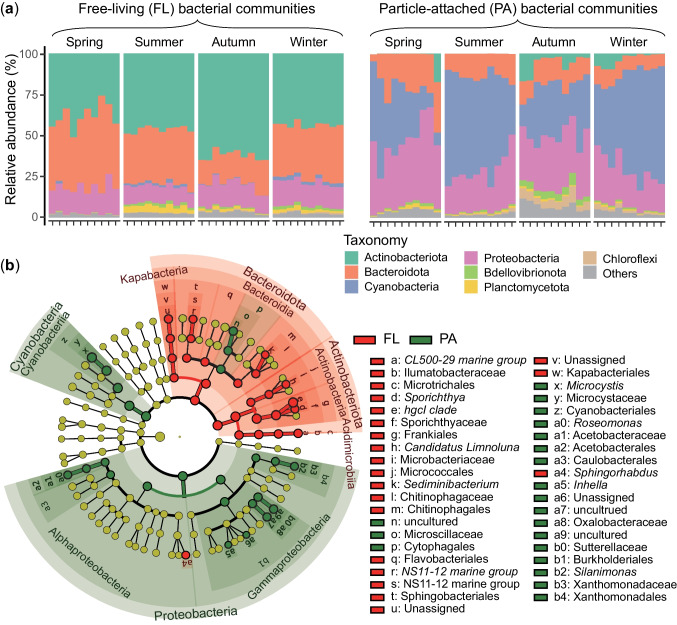
**a** Relative abundance of the dominant phyla detected in free-living (FL) and particle-attached (PA) bacterial communities at each sampling site across seasons in Lake Xingyun. Samples from Site 1 to Site 10 are presented along the x-axis in each panel. **b** LEfSe results illustrating taxonomic differences in bacterial communities between FL and PA fractions. Red and green circles represent those bacterial taxa significantly enriched in FL and PA fractions, respectively, while yellow circles represent taxa with nonsignificant differences between the two fractions

Lake Xingyun’s FL and PA bacteria displayed distinct taxonomic compositions (Fig. 5a). At the phylum level, the FL community was primarily composed of Actinobacteria (47.6%), Bacteroidota (31.7%), and Proteobacteria (13.8%). In contrast, the PA bacterial community was predominantly composed of Cyanobacteria (43.8%), Proteobacteria (30.3%), and Bacteroidota (14.1%). At the genus level, the FL community was mainly composed of the *hgcI clade* (30.7%), *CL500-29 marine group* (9.7%), *Flavobacterium* (7.4%), and an unclassified genus from the class Kapabacteria (5.0%). Meanwhile, the PA bacterial community was predominantly composed of *Microcystis* (37.8%), three unclassified genera from the family Microscillaceae (7.3%), family Sutterellaceae (6.3%), and family Oxalobacteraceae (4.5%) (Fig. S6). Significant seasonal variations in taxonomic composition were presented in both PA and FL fractions, with the PA fraction showing more pronounced variations in the relative abundance of major taxa (Fig. 5a). Additional analysis using LEfSe unveiled significant differences in 48 bacterial taxa between FL and PA fractions (Fig. 5b). Most of the dominant taxa in FL and PA bacteria showed significant preferences for different fractions.

### Environmental Drivers on Bacterial Community Variations

**Fig. 6:**
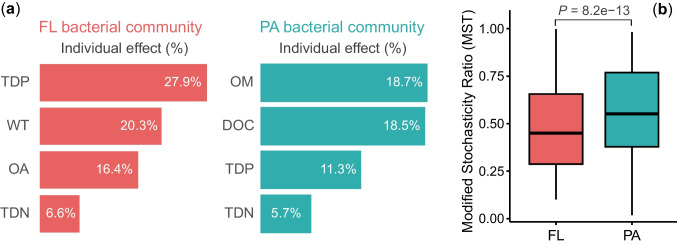
Ecological processes governing free-living (FL) and particle-attached (PA) bacterial communities. **a** The relative contribution (individual effect) of each environmental variable significantly related to variations in FL and PA bacterial communities. Results were obtained from redundancy analyses ordination with forward selection. **b** Modified stochasticity ratio (MST) estimating stochasticity in FL and PA bacterial community assembly

RDA revealed that the seasonal variations in BCCs within the FL fraction were primarily driven by TDP, WT, A_OA_, and TDN, collectively explaining 71.1% of the total variation (adjusted *R*^2^). Notably, TDP emerged as the most influential individual factor, solely accounting for 27.9% of the total variation (Fig. 6a).

Similarly, the variations in BCCs across seasons within the PA fraction were attributed to OM, DOC, TDP, and TDN, jointly explaining 54.2% of the total variation. Of these environmental parameters, OM emerged as the most important contributor, independently explaining 18.7% of the total variation (Fig. 6a).

### Assembly Mechanisms of FL and PA Bacterial Communities

Findings from MST analysis revealed distinct ecological processes shaping FL and PA bacterial communities. Specifically, FL communities demonstrated a stronger influence of deterministic processes (MST = 0.49) compared to stochastic processes. Conversely, the assembly of PA communities showed a higher degree of stochasticity (MST = 0.57) (Fig. 6b). Importantly, the observed differences in community assembly between FL and PA fractions were statistically significant (*P* < 0.001).

### Co-occurrence Networks of FL and PA Bacterial Communities

**Fig. 7:**
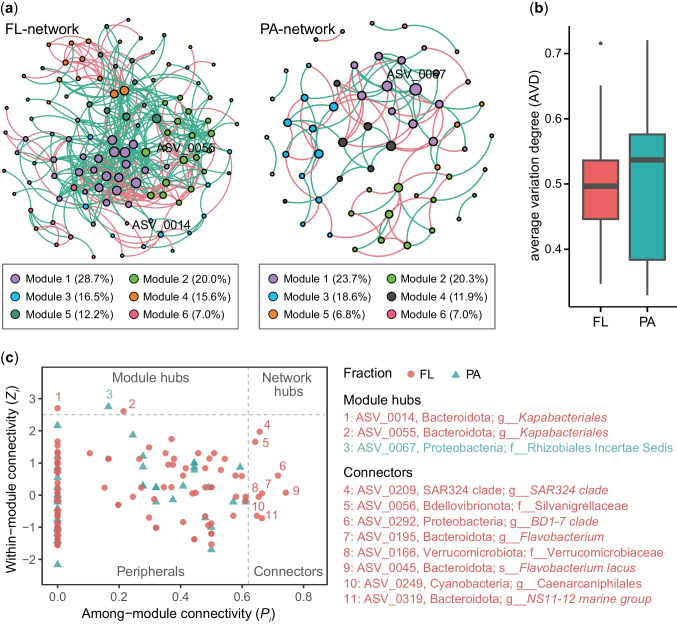
**a** Co-occurrence networks and their modular properties for free-living (FL) and particle-attached (PA) bacterial communities in Lake Xingyun. ASVs were selected based on their relative abundance (> 0.05%) and occurrence (in > 50% samples). The size of each node is proportional to the number of connections (degree), and nodes are colored based on different modules. Green and red edges indicate positive and negative correlations, respectively. **b** Average variation degree (AVD) of FL and PA bacterial communities. **c** Visualization of node categories. Each symbol represents an ASV from bacterial communities in FL and PA fractions. The topological role of each ASV (node) was determined based on the scatter plot of within-module connectivity (*Z*_i_) and among-module connectivity (*P*_i_). Nodes in the network are categorized as peripheral nodes (*Z*_i_ < 2.5, *P*_i_ < 0.62), connectors (*Z*_i_ < 2.5, *P*_i_ ≥ 0.62), module hubs (*Z*_i_ ≥ 2.5, *P*_i_ < 0.62), and network hubs (*Z*_i_ ≥ 2.5, *P*_i_ ≥ 0.62)

The results of network analysis revealed distinct co-occurrence patterns within FL and PA bacterial communities. FL bacterial networks consisted of 115 nodes with 468 edges, whereas PA bacterial networks comprised 59 nodes with 110 edges (Fig. 7a; Table 1). Higher average clustering coefficients (avgCC) and average path lengths (APL) in FL and PA bacterial networks compared to their corresponding random networks indicate the presence of “small-world” properties. This observation is further supported by small-world coefficients (σ) of 49 for FL and 124 for PA bacterial networks, with σ > 1 indicating their “small-world” nature [47].

**Table 1:** Topological properties of the co-occurrence networks analyzed for abundant (relative abundance > 0.05%) and frequent ASVs (present in more than half of the samples) in both PA and FL fractions. Additionally, identically sized Erdős-Rényi random networks were constructed for comparison

	Empirical network									Random network	
	Nodes	Edges		Modularity	avgK	AvgCC	APL	E	σ	AvgCC	APL
		Positive	Negative								
FL	115	174 (37.2%)	294 (62.8%)	0.468	8.14	0.378	3.34	0.37	49	0.006 ± 0.009***	2.58 ± 0.03***
PA	59	61 (55.4%)	49 (44.6%)	0.586	3.73	0.271	3.39	0.38	124	0.002 ± 0.008***	3.10 ± 0.12***

Student’s *t* tests were employed to discern the differences in network properties between the empirical and random networks

*PA* particle-attached, *FL* free-living, *avgK* average degree, *avgCC* average clustering coefficient, *APL* average path length, *E* geodesic efficiency, *σ* small-word coefficient, indicates high interconnectivity and high efficiency (Telesford et al., 2011)

^***^P < 0.001. The differences of modularity, avgK, AvgCC, and APL between FL and PA fractions were also detected using Student’s *t* tests. Results showed that the differences of them are all significant (*P* < 0.001)

The average degree (avgK) and avgCC were notably higher in the FL bacterial network compared to the PA network (Table 1), indicating a greater number of interactions among species and a more complex network structure within FL bacterial communities than in their PA counterparts. Furthermore, the co-occurrence network in the FL fraction exhibited predominantly negative relationships (62.8%), suggesting ecological competition within the FL microbiome. In contrast, the PA bacterial network displayed a higher proportion of positive correlations among edges (55.4%), emphasizing the influence of mutualistic or cooperation interactions in the PA bacterial community assembly. The AVD value was lower in FL bacterial communities compared to their PA counterparts (Fig. 7b), indicating higher stability within FL bacterial communities.

Modularity analysis unveiled distinct patterns in the organization of nodes within the networks. In the PA network, all ASVs were identified as peripherals, with the exception of one ASV (ASV_0067) classified as a module hub (Fig. 7c). In contrast, the FL network exhibited two module hub nodes and eight connectors (Fig. 7c). Notably, both module hub nodes are associated with the genus *Kapabacteriales* of the Bacteroidota phylum. Additionally, among the eight connectors, two were classified as belonging to the genus *Flavobacterium* within Bacteroidota.

## Discussion

### Differences in PA and FL Bacterial α-Diversities Across Fractions and Seasons

This study unveils a significant and intriguing observation, as depicted in Fig. 2a, wherein α-diversity indices within FL communities were notably higher than those within PA counterparts in the hypereutrophic Lake Xingyun. This departure from trends observed in prior studies, such as those in oligotrophic Tibetan lakes [48], mesotrophic Lake Tianmu [23], Lake Poyang [49], mesotrophic to eutrophic Xidong Reservoir [13], eutrophic Lake Taihu [12, 18], and eutrophic Lake Wuliangsuhai [15], which consistently reported higher *α*-diversity in PA bacteria compared to their FL counterparts, signifies a unique ecological context. The apparent discrepancy in *α*-diversity trends between FL and PA fractions may be trophic-dependent, as nutrient availability plays a pivotal role in determining bacterial compositions, organic particle characteristics, and interactions between bacteria and phytoplankton.

A comprehensive comparative analysis conducted by Xie et al. [21] across lakes with different nutrient levels revealed increasing differences in Chao1 richness between FL and PA bacteria from eutrophic to oligotrophic conditions. In the oligotrophic Lake Fuxian, the Chao1 richness of PA bacteria was 3.3 times that of FL bacteria, whereas in the eutrophic Lake Taihu, the Chao1 richness of PA bacteria was only 1.3 times that of FL bacteria. However, the Shannon index between PA and FL communities was not significant in eutrophic Lake Taihu. Additionally, a study encompassing various regions in Lake Taihu demonstrated that differences in *α*-diversity indices between PA and FL fractions were insignificant in the middle-eutrophic river mouth area [16]. Conversely, significantly higher *α*-diversity indices (species richness and Shannon index) were noted in the FL fraction compared to the PA counterpart in areas experiencing cyanobacterial blooms. These collective findings, alongside the results of this study, underscore the effect of nutrient levels on bacterial diversity patterns between FL and PA fractions in freshwater lakes. Specifically, in oligotrophic lakes, the PA fraction harbors more diverse bacteria, and the differences in bacterial diversity between the two fractions decrease with increasing nutrient levels. In hypereutrophic lakes dominated by cyanobacteria, the FL community exhibits higher bacterial diversity compared to the PA fraction.

Two factors may account for the higher α-diversity in the FL bacterial fraction compared to the PA counterpart in hypereutrophic lakes. Firstly, nutrient availability plays a crucial role; FL bacteria may benefit from the elevated nutrient levels in hypereutrophic lakes, such as increased concentrations of nitrogen and phosphorus. These nutrients can support the growth of a diverse range of planktonic species [50, 51]. Under hypereutrophic conditions, FL bacteria in the water column are less likely to lack nutrients compared to PA counterparts dwelling on nutrient-abundant organic particles [6]. Conversely, in oligotrophic lakes, limited nutrient availability may constrain the improvement of FL bacterial diversity. Secondly, microenvironmental heterogeneity plays a role; FL bacteria experience a more dynamic and heterogeneous microenvironment in the water, promoting the coexistence of diverse species. In contrast, PA bacteria in hypereutrophic lakes may encounter more stable and localized conditions, leading to a selective advantage for specific species and potentially lower overall diversity. For instance, in hypereutrophic Lake Xingyun, particles mainly originate from cyanobacterial particles (Cyanobacteria constitute more than 94% of the total phytoplankton cell density), especially *Microcystis* spp. (dominated in every season) [52], resulting in a lack of compositional and microhabitat diversity [27] and a subsequent reduction in the diversity of attached bacteria [53]. This aligns with the significantly narrower ecological niche of PA bacteria compared to FL bacteria (Fig. 4c), indicating lower available bioresource diversity for the PA environment compared to the water column.

Our study further revealed significant seasonal variations in the *α*-diversity of both PA and FL bacteria in hypereutrophic Lake Xingyun (Fig. 2b). Similar seasonal variation patterns have been observed in various lakes, including oligotrophic Lake Fuxian [21], mesotrophic Lake Tianmu [23], Lake Tiefwaren [54], the seasonally flooded Lake Poyang [49], eutrophic Lake Taihu [18], and the Aulne estuary [24]. Interestingly, during spring and summer, marked by elevated cyanobacterial blooms (as indicated by Chl-*a* concentration, Fig. S1), there was a significant decrease in both richness and the PD index of PA bacteria compared to autumn and winter. However, the richness and PD index of FL bacteria in the summer were only surpassed by those in the winter (Fig. 2b). In line with these results, a prior investigation in eutrophic Lake Mochou revealed a decrease in the α-diversity of the PA community during cyanobacterial bloom periods, while that of the FL counterpart increased [55]. These collective observations underscore the profound impact of cyanobacterial blooms on *α*-diversity patterns in both FL and PA bacteria [56]. Prior research has suggested that healthy *Microcystis* inhibits the attachment of bacteria, as observed by scanning electric microscopy and epifluorescence microscopy [16, 57], while secretions released by Cyanobacteria can promote the growth of plankton bacterial diversity [58, 59]. Thus, the concept that particles play a vital role as hotspots for bacterial diversity, as previously proposed by Grossart [10], seems to be inapplicable in hypereutrophic lakes suffering from heavy cyanobacterial blooms. Homogeneous particles with high cell activities, such as *Microcystis* colonies, decrease the diversity of attached bacteria.

### Dynamics of PA and FL Bacterial Communities and the Driving Factors

The composition of PA and FL bacterial communities displayed distinct characteristics in Lake Xingyun (Fig. 5 and Fig. S6). The dominance of Cyanobacteria in the PA fraction aligns with findings from various freshwater [21, 54] and marine ecosystems [60]. In culture conditions, Proteobacteria were predominant in the colony-attached bacterial community, while Bacteroidetes were prevalent in the FL community [61]. At the genus level, *Roseomonas* has previously been identified as a prominent component associated with *Microcystis aeruginosa* cell surfaces [62], cyanobacterial blooms [63], and > 20-µm particles in Lake Kinneret [64]. *Silanimonas*, represented by a novel species (*S. algicola*) isolated from *Microcystis* colonies [65], possesses nitrogen removal capabilities as autotrophic denitrifies [66, 67]. These findings suggest a potential specialization of these genera in particle-associated environments, particularly within cyanobacterial colonies in freshwater ecosystems.

The genera *CL500-29 marine group* (i.e., *acIV*) and *hgcI clade* (*acI*) are recognized as common freshwater genera and have been consistently identified as dominant in FL bacterial communities [16, 51, 68, 69]. Planktonic bacterial genera, such as *NS11-12 marine group* and *Sediminibacterium*, have been abundantly found in shallow freshwater lakes [16, 70–72]. During the spring, there was an elevated relative abundance of the *Flavobacterium* genus observed in the FL fraction (Fig. S6) when high cyanobacterial blooms (indicated by Chl-*a*) coincided with the highest concentrations of DOC (Fig. S1). Actinobacteria- and Bacteroidota-related genera are often detected in Cyanobacteria-dominated freshwater ecosystems and have the ability to utilize DOC produced by Cyanobacteria blooms or released after their demise [58, 73, 74].

Both FL and PA bacterial communities exhibited significantly seasonal succession patterns, although the driving factors exhibited both similarities and differences (Figs. 3 and 6). Concentrations of dissolved phosphorus and nitrogen demonstrated strong correlations with both FL and PA assemblages, with additional influences of WT and OA abundance shaping FL community variations, and OM proportion and DOC concentrations significantly regulating PA bacterial variations. This implies that while both fractions share certain environmental interactions, there are distinct mechanisms at play in their responses to environmental parameters.

Dissolved nutrients, particularly TDP and TDN, act as crucial limiting factors influencing the proliferation of both bacteria and phytoplankton [75], thereby shaping the seasonal dynamics in bacterial communities through direct effects and indirect interactions between phytoplankton and bacteria [76]. Water temperature directly and indirectly influences the seasonal variations in bacterial assemblages and has been thoroughly discussed in literature [14, 18, 76, 77]. OA abundance emerged as a key driver for the variations observed in FL bacterial communities (Fig. 6a), underscoring the ecological interactions between particles and FL bacteria. Heavily colonized organic particles can act as “baby machines,” releasing attached bacteria into the surrounding water column [78, 79].

The proportion of OM stands out as a pivotal factor influencing the dynamics of PA bacterial assemblages in Lake Xingyun (Fig. 6a). Higher proportions of OM were observed during spring and summer due to more substantial cyanobacterial blooms (Fig. S2). PA bacteria play a crucial role in POM mineralization through the secretion of multiple extracellular hydrolases [80, 81]. The synthesis and activity of these adaptative extracellular enzymes are regulated by the organic substrates to some extent. During season succession, changes not only in the proportion of OM but also in the physiological and biochemical states of the organic matter (live phytoplankton cells or dead detritus) influence the community structures of PA bacteria. As POM degrades, DOC is released from the surface of OAs, serving as sources of carbon and energy for heterotrophic bacteria. OAs in the water column can create a comet-shaped eutrophic plume of DOC, with the majority still bound to particle surfaces [80, 82]. Consequently, the concentration and composition of DOC impact the seasonal patterns of PA bacterial assemblages [83]. In turn, the dynamics of PA bacteria also influence the DOC pool existing in lake water and the FL bacteria dwelling within it.

### Overlap Between PA and FL Bacterial Communities

This study showed that 41.0% of the total ASVs, representing 89.9% of the total reads, were shared between both FL and PA bacterial fractions (Fig. 4). This substantial overlap suggests a significant exchange of bacterial taxa (ASVs) between FL and PA lifestyles or active shifts among these fractions. The presence of highly overlapping bacterial species in both fractions has also been observed in various water bodies such as Lake Esrum [79], Lake Stechlin [22], Lake Taihu [84], Xidong Reservoir [13], and Lake Wuli [17], emphasizing the ecological connectivity between these fractions. These findings underscore the capability of a large proportion of bacterial cosmopolitan species (generalists) to inhabit both dissolved and particulate habitats in natural water bodies [85]. Bacterial communities in aquatic ecosystems, both FL and PA, appear to engage in interactive assemblages [79].

A prior study reported a higher overlap, with 59% of the total species occupying 96.4% of the total reads shared between FL and PA fractions in eutrophic shallow Lake Taihu [14], exceeding the overlap observed in this study. The extent of overlap is influenced by particulate matter characteristics (related to lake eutrophic status and exogenous input) and hydrodynamic conditions [14, 79]. In Lake Xingyun, the majority of particulate matter in the water column, owing to eutrophication, originates from cyanobacteria, potentially recruiting specific bacterial taxa and thereby reducing overlap between FL and PA communities. This reduction in the relative abundance of shared ASVs during high cyanobacterial bloom seasons (spring and summer) compared to low bloom seasons (autumn and winter) supports this observation (Fig. 4b, Fig. S1). Furthermore, the larger size and shallower nature of Lake Taihu, along with enhanced hydrodynamic disturbance, contribute to increased exchanges between PA and FL communities. This finding is in line with our previous results, indicating that the proportions of shared species between PA and FL fractions in the river mouth of Lake Taihu (experiencing strong hydrodynamic disturbance) were significantly higher compared to those observed in areas affected by cyanobacterial blooms [16].

### Co-occurrence Patterns and Stability of PA and FL Bacterial Communities

Network analysis uncovered that the FL network in Lake Xingyun exhibited greater stability than the PA network, as evidenced by significantly higher avgK, avgCC, and increased proportion of negative correlations among edges in the FL network (Table 1). The higher values of avgK and avgCC in the FL network suggest an increased number of ecological connections and greater community complexity within the FL bacterial community [39]. The lower AVD value in FL bacterial communities compared to the PA counterpart further confirmed the robustness of higher stability of FL bacterial communities (Fig. 7b). This finding contrasts with previous studies in Lake Tianmu [23] and Lake Taihu [18], which suggested that the stability of PA community surpassed that of FL counterpart. Several factors may explain this discrepancy. Firstly, in this study, the higher stability of FL bacterial communities may be attributed to their higher *α*-diversity. In mesotrophic Lake Tianmu and eutrophic Lake Taihu, PA bacteria demonstrated significantly higher *α*-diversities than those in FL fractions [18, 23]. Communities with high *α*-diversity are likely more resistant to environmental disturbance due to comprising multiple species with redundant functions, providing a buffer against the loss of individual species [86–88].

Secondly, the nutrient-rich environment in the water column of Lake Xingyun may make FL bacteria less susceptible to nutrient limitation, potentially enhancing the stability of FL bacterial communities. In oligotrophic, mesotrophic, and even eutrophic lakes, particle-associated habitat is considered “hot spots” for nutrients and is likely to harbor a broader array of microhabitats available to bacteria with a PA lifestyle [5, 89]. However, in hypereutrophic Lake Xingyun, the elevated concentrations of dissolved nitrogen, phosphorus, and DOC (Fig. S1) could supply abundant resources for FL bacteria, contributing to the stability of the community. Additionally, the prevailing dominance of Cyanobacteria as the primary source of organic particles in Lake Xingyun, coupled with the effects of eutrophication, may contribute to a decreased resistance of PA bacterial communities to environmental perturbations compared to the FL bacterial fraction. This is attributed to the adverse effects exerted by healthy cyanobacterial colonies on PA bacteria [57]. Moreover, the presence of homogeneous cyanobacterial detritus inhibits the development of bacterial diversity with an attached lifestyle [53].

Thirdly, the increased complexity of the FL network may contribute to the stability of FL bacterial assemblages. In soil and lake ecosystems, there have been observed positive correlations between microbial network complexity and stability [90, 91]. However, conflicting reports of negative correlations between complexity and stability have also been documented [18, 92]. The relationship between complexity and stability in microbial communities is proposed to be regulated by community initial diversity and the interaction strengths between species [87, 92]. In this study, a significantly larger percentage of negative correlations among edges (62.8%) was found in the FL network compared to the PA network (44.6%), suggesting a greater degree of species competition in FL bacterial community assembly. Model predictions have suggested that a high species diversity is more likely to coexist stably when the microbial community is dominated by competitive rather than cooperative interactions [93]. Moreover, the discovery of additional keystone taxa, such as module hubs, connectors, and network hubs, in the FL network (Fig. 7c) aligns with the results of Liu et al. [91], who reported that networks containing more keystone taxa typically demonstrate stability in eutrophic Lake Donghu.

## Conclusions

This study is the first to explore the spatiotemporal variations of bacterial diversity, composition, assembly processes, and stability within both particle-attached (PA) and free-living (FL) bacterial assemblages inhabiting the hypereutrophic Lake Xingyun, marked by intense cyanobacterial blooms. Our observations revealed pronounced seasonal fluctuations in environmental parameters, exerting significant influence on bacterial community structures. Specifically, variations in FL communities were chiefly steered by factors like total dissolved phosphorus (TDP), water temperature (WT), organic aggregate abundance, and total dissolved nitrogen (TDN). On the other hand, particulate-attached communities showed a distinct response, where organic matter proportion (OM), dissolved organic carbon (DOC), TDP, and TDN played pivotal roles in governing their dynamics. Bacterial communities in the FL fraction displayed higher α-diversity than their PA counterparts, accompanied by discernible taxonomic compositions. The FL network demonstrated greater stability, complexity, and negative interactions, indicative of competitive relationships. Conversely, the PA network displayed a prevalence of positive correlations, suggesting mutualistic interactions. Substantial overlap between PA and FL bacterial species underscored the ecological connectivity between them. Importantly, our findings deviate substantially from those observed in oligotrophic, mesotrophic, and eutrophic lakes. This disparity can be attributed, at least in part, to the elevated nutrient levels and the dominance of organic particles originating from cyanobacteria in Lake Xingyun. Altogether, our study elucidates the complex interplay among environmental factors, bacterial fractions, and seasonal dynamics, offering insights into the cascading effects of nutrient status on the ecological processes shaping bacterial communities in freshwater lake ecosystems.

### Supplementary Information

Below is the link to the electronic supplementary material.

Supplementary file1 (DOCX 1503 KB)

## Data Availability

The raw sequence data mentioned in this paper have been deposited in the Genome Sequence Archive at the National Genomics Data Center, China National Center for Bioinformation (accession number: CRA014578), and are publicly accessible at https://ngdc.cncb.ac.cn/gsa.
