# Supplementary material for: Beyond the Bloom: Unraveling the Diversity, Overlap, and Stability of Free-Living and Particle-Attached Bacterial Communities in a Cyanobacteria-Dominated Hypereutrophic Lake
Source: Microb Ecol. 2024 Jul 24;87:96. doi: 10.1007/s00248-024-02410-2 (PMC11269507; doi:10.1007/s00248-024-02410-2)
Supplement: Supplementary file 1 [file 248_2024_2410_MOESM1_ESM.docx]

*Supplementary Materials*

# Beyond the bloom: unraveling the diversity, overlap and stability of free-living and particle-attached bacterial communities in a cyanobacteria-dominated hypereutrophic lake

Guijuan Xie^1, 2^, Chuanbo Sun^1^, Yi Gong ^2^, Wenlei Luo^2,3^, Xiangming Tang^2,4*^

^1^ College of Biology and Pharmaceutical Engineering, West Anhui University, Lu’an 237012, China

^2^ State Key Laboratory of Lake Science and Environment, Nanjing Institute of Geography and Limnology, Chinese Academy of Sciences, Nanjing 210008, China

^3^ The Fuxianhu Station of Plateau Deep Lake Field Scientific Observation and Research, Yunnan, Yuxi 653100, China

^4^ College of Resources and Environment, University of Chinese Academy of Sciences, Beijing 100049, China

*** Corresponding author:**

Xiangming Tang

xmtang@niglas.ac.cn

**Contents of this file:**

Figure S1-S5

**Fig. S1.** Comparative analysis of the primary environmental parameters across various seasons in Lake Xingyun. WT: water temperature; SD: Secchi disk of transparency; TN: total nitrogen; TDN: total dissolved nitrogen; TP: total phosphorus; TDP: total dissolved phosphorus; COD: the permanganate index; DOC: dissolved organic carbon; Chl-*a*: chlorophyll*-a*; TSS: total suspended solids; TLI: trophic level index. Kruskal-Wallis test was conducted to assess differences across seasons. Different italic lower-case letters at the top of each boxplot indicate significant differences (*P* < 0.05). In the boxplot, bold short black lines and yellow dots represent the median and mean of each parameter in each season, respectively.

**Fig. S2.** Comparison of the main parameters related to organic aggregates (OA) among different seasons in Lake Xingyun. PAB: OA-attached bacterial abundance; FLB: free-living bacterial abundance; TB: total bacterial abundance; OM: percentage of organic matter in total suspended solids; PN: particle-related nitrogen; TN: total nitrogen; PP: particle-related phosphorous; TP: total phosphorous.

**Fig. S3.** The rarefaction curves for the observed features, i.e., Amplicon Sequence Variants (ASVs), demonstrating their distribution across different fractions, seasons, and sampling sites. These curves approach an asymptotic maximum once the sequencing depth exceeds 10,000, which suggests that the sequencing depth is adequate to provide a stable and unbiased estimation of species richness.

**Fig. S4.** Boxplots illustrating the α-diversity indices (Shannon index and Pielou evenness) across seasons for (**a**) free-living (FL) and (*b*) particle-attached (PA) bacterial communities in Lake Xingyun. Diversity indices were computed using a subset of 33,669 sequences per sample.

**Fig. S5.** Boxplots of the α-diversity indices among sampling sites for (**a**) free-living (FL) and (**b**) particle-attached (PA) bacterial assemblages in Lake Xingyun. Diversity indices were calculated using subset of 33,669 sequences per sample.

**Fig. S6.** The relative abundance of dominant genera detected in free-living (FL) and particle-attached (PA) bacterial communities at each sampling site across seasons in Lake Xingyun. In each panel, *x*-axis represents samples from Site 1 to Site 10.

**Table S1** Summary of the numbers of reads for each sample in this study. PA: particle-attached; FL: free-living; QC: quality control.

| SampleID | Fraction | Sampling site | Raw Reads | Post-QC | Final reads |
| --- | --- | --- | --- | --- | --- |
| XY01A01 | PA | 1 | 68738 | 48937 | 44230 |
| XY01A04 | PA | 1 | 69469 | 47501 | 40603 |
| XY01A07 | PA | 1 | 69114 | 53048 | 49729 |
| XY01A11 | PA | 1 | 68171 | 47259 | 37637 |
| XY01F01 | FL | 1 | 68461 | 38382 | 37276 |
| XY01F04 | FL | 1 | 68446 | 35723 | 35710 |
| XY01F07 | FL | 1 | 69279 | 38524 | 38489 |
| XY01F11 | FL | 1 | 69141 | 35711 | 35567 |
| XY02A01 | PA | 2 | 68897 | 49146 | 44592 |
| XY02A04 | PA | 2 | 69538 | 52235 | 50845 |
| XY02A07 | PA | 2 | 69256 | 52594 | 51398 |
| XY02A11 | PA | 2 | 68523 | 46274 | 34989 |
| XY02F01 | FL | 2 | 68683 | 40220 | 39199 |
| XY02F04 | FL | 2 | 68562 | 35240 | 35153 |
| XY02F07 | FL | 2 | 68355 | 37757 | 37696 |
| XY02F11 | FL | 3 | 69276 | 34244 | 34154 |
| XY03A01 | PA | 3 | 69136 | 48224 | 44082 |
| XY03A04 | PA | 3 | 69669 | 48608 | 46488 |
| XY03A07 | PA | 3 | 69396 | 50619 | 49022 |
| XY03A11 | PA | 3 | 68619 | 46915 | 34171 |
| XY03F01 | FL | 3 | 68905 | 41392 | 40717 |
| XY03F04 | FL | 3 | 68683 | 33711 | 33669 |
| XY03F07 | FL | 3 | 68656 | 37621 | 37520 |
| XY03F11 | FL | 3 | 69501 | 36126 | 35768 |
| XY04A01 | PA | 4 | 69211 | 49216 | 43209 |
| XY04A04 | PA | 4 | 69974 | 51441 | 48925 |
| XY04A07 | PA | 4 | 69590 | 51939 | 50563 |
| XY04A11 | PA | 4 | 68650 | 47858 | 36810 |
| XY04F01 | FL | 4 | 68930 | 40919 | 39728 |
| XY04F04 | FL | 4 | 68942 | 35622 | 35571 |
| XY04F07 | FL | 4 | 68791 | 37362 | 37267 |
| XY04F11 | FL | 4 | 70324 | 34274 | 34153 |
| XY05A01 | PA | 5 | 69361 | 51395 | 47745 |
| XY05A04 | PA | 5 | 69045 | 49224 | 46813 |
| XY05A07 | PA | 5 | 69661 | 50114 | 48605 |
| XY05A11 | PA | 5 | 68882 | 47728 | 33755 |
| XY05F01 | FL | 5 | 68206 | 39521 | 37876 |
| XY05F04 | FL | 5 | 69041 | 36203 | 36164 |
| XY05F07 | FL | 5 | 68923 | 36663 | 36531 |
| XY05F11 | FL | 5 | 69581 | 37527 | 37376 |
| XY06A01 | PA | 6 | 68558 | 49906 | 45635 |
| XY06A04 | PA | 6 | 70314 | 48579 | 46578 |
| XY06A07 | PA | 6 | 68782 | 50061 | 48543 |
| XY06A11 | PA | 6 | 69008 | 46070 | 34944 |
| XY06F01 | FL | 6 | 68229 | 40071 | 38007 |
| XY06F04 | FL | 6 | 69175 | 34906 | 34860 |
| XY06F07 | FL | 6 | 69081 | 36361 | 36289 |
| XY06F11 | FL | 6 | 68831 | 38956 | 38457 |
| XY07A01 | PA | 7 | 68748 | 53274 | 51360 |
| XY07A04 | PA | 7 | 68221 | 42970 | 41644 |
| XY07A07 | PA | 7 | 68863 | 53448 | 53325 |
| XY07A11 | PA | 7 | 69182 | 46377 | 35732 |
| XY07F01 | FL | 7 | 68283 | 42532 | 40478 |
| XY07F04 | FL | 7 | 68281 | 34419 | 34390 |
| XY07F07 | FL | 7 | 69145 | 37641 | 37427 |
| XY07F11 | FL | 7 | 69899 | 35129 | 34875 |
| XY08A01 | PA | 8 | 68798 | 49329 | 46738 |
| XY08A04 | PA | 8 | 68311 | 43049 | 41700 |
| XY08A07 | PA | 8 | 68129 | 50568 | 49409 |
| XY08A11 | PA | 8 | 68206 | 46638 | 35508 |
| XY08F01 | FL | 8 | 68451 | 39713 | 36482 |
| XY08F04 | FL | 8 | 68292 | 36212 | 36194 |
| XY08F07 | FL | 8 | 69360 | 37504 | 37177 |
| XY08F11 | FL | 8 | 69113 | 38765 | 37635 |
| XY09A01 | PA | 9 | 68850 | 53076 | 51095 |
| XY09A04 | PA | 9 | 68438 | 42056 | 41552 |
| XY09A07 | PA | 9 | 68281 | 48292 | 46953 |
| XY09A11 | PA | 9 | 68369 | 47990 | 41947 |
| XY09F01 | FL | 9 | 68489 | 39790 | 36945 |
| XY09F04 | FL | 9 | 68463 | 37189 | 37132 |
| XY09F07 | FL | 9 | 69486 | 37799 | 37117 |
| XY09F11 | FL | 9 | 70314 | 35657 | 35621 |
| XY10A01 | PA | 10 | 68989 | 53747 | 50028 |
| XY10A04 | PA | 10 | 68488 | 40413 | 39433 |
| XY10A07 | PA | 10 | 68350 | 45421 | 43854 |
| XY10A11 | PA | 10 | 70794 | 45530 | 39495 |
| XY10F01 | FL | 10 | 68750 | 39426 | 35624 |
| XY10F04 | FL | 10 | 68622 | 35579 | 35543 |
| XY10F07 | FL | 10 | 68584 | 36635 | 36493 |
| XY10F11 | FL | 10 | 70371 | 37213 | 37093 |
| **Sum** |  |  | **5516484** | **3441308** | **3239107** |
